# Clinical Features of Long COVID Patients Coinfected With *Mycoplasma pneumoniae*

**DOI:** 10.1155/cjid/7213129

**Published:** 2024-12-07

**Authors:** Xiaodan Zhu, Yanhua Li, Jinghua Wang, Weifei Gao

**Affiliations:** ^1^Department of General Practice, The Affiliated Hospital of Hangzhou Normal University, Hangzhou 310000, China; ^2^Department of General Practice, The Second Affiliated Hospital Zhejiang Chinese Medical University, Hangzhou 310000, China; ^3^Department of General Practice, Third People's Hospital of Hangzhou, Hangzhou 310000, China

**Keywords:** clinical characteristic, coinfection, coronavirus disease 2019, long COVID, *Mycoplasma pneumoniae*

## Abstract

**Background:** Since the SARS-CoV-2 pandemic, many patients have suffered prolonged complications, called “long COVID.” *Mycoplasma pneumoniae* is a common respiratory pathogen. Reports of simultaneous long COVID and *M. pneumoniae* infections are rare in the literature.

**Methods:** We analyzed the clinical data of patients with long COVID-19 who visited the Respiratory Clinic of The Affiliated Hospital of Hangzhou Normal University between January 1 and January 31, 2023, together with their laboratory and radiographic findings, with Pearson's *χ*^2^ test.

**Results:** Fifty-two patients diagnosed with both long COVID and *M. pneumoniae* infection and 77 with long COVID only were compared. The ages, clinical symptoms, and comorbidities of the two groups did not differ significantly (*p* > 0.05). However, sex and imaging findings differed between the groups.

**Conclusions:** Our study showed that long COVID–*M. pneumoniae* coinfection was more commonly seen in females and patients with typical chest computed tomography (CT) images.

## 1. Introduction

By February 9, 2023, more than 755 million SARS-CoV-2 (COVID-19) infections and over 6.8 million COVID-associated deaths worldwide had been reported by the World Health Organization (WHO). Evidence quickly emerged that patients infected with COVID-19 not only experienced high levels of morbidity and mortality but also many (almost 70% of COVID-19 survivors) suffered a long period of complications. In October 2021, the term “long COVID” was introduced by WHO [1, 2] and applied to the persistent symptoms of COVID-19 survivors. According to WHO, symptom onset occurs during or up to more than 12 weeks after SARS-CoV-2 infection and can be explained by no alternative diagnosis. However, some experts consider that patients with long-term symptoms lasting 2 weeks have a mild disease and those with symptoms lasting 4 weeks have moderate-to-severe illness, whereas critically ill patients with symptoms lasting 6 weeks or more have “long COVID” syndrome [3]. Fatigue, cough, chest pain, dyspnea, palpitations, arthralgia, muscle pain, cognitive impairment, and anxiety are the main manifestations in patients with long COVID, and they impose a substantial burden on the COVID survivor's quality of life.

COVID-19 outbreaks often occur at the time of the year when other respiratory infections are most common, and coinfections with other respiratory pathogens are a common feature of pandemics. *Mycoplasma pneumoniae* is a common atypical bacterium among respiratory pathogens [4, 5], and infections can occur in patients of all ages. It generally causes a mild self-limiting disease [6, 7]. However, when long COVID is complicated by coinfection with *M. pneumoniae*, the treatment of COVID-19 becomes more difficult. The characteristics of patients with both long COVID and *M*. *pneumoniae* infection must be clarified to allow their clinical management. Long COVID complicated with *M*. *pneumoniae* infection is rarely documented in the medical community. A meaningful description of the clinical characteristics of patients with long COVID and *M. pneumoniae* coinfection, including laboratory results and radiological findings, is the aim of this study [8].

## 2. Methods

In this study, we retrospectively collected data on the presentations of long COVID patients, their demographic details, and test results from electronic records (laboratory data and chest computed tomography [CT] findings). Patients underwent the following laboratory tests: complete blood counts, C-reactive protein, creatine kinase, lactate dehydrogenase, and transaminase. The patients were diagnosed with long COVID and/or *M. pneumoniae* between January 1 and January 31, 2023, at the Respiratory Clinic at The Affiliated Hospital of Hangzhou Normal University (Hangzhou City, Zhejiang Province, China).

After the initial detection of COVID-19 antigen, the viral load becomes undetectable [9]. This marks the start of long COVID. Presentation with acute respiratory symptoms, such as fever and cough, and no viral antigen after its initial detection were used as the diagnostic criteria for long COVID. The diagnostic criterion for detecting *M. pneumoniae* infections involves utilizing a method from a Japanese antibody test kit designed to identify MP-IgM antibodies specific to *M*. *pneumoniae*. These antibodies can typically be detected 5–6 days after infection. A titer of 1:160 or higher is considered positive. A positive result suggests a possible *M. pneumoniae* infection, while a negative result does not completely rule out MP infection. And this method is particularly suitable for rapid screening. To confirm *M. pneumoniae* infection, it is essential to conduct a comprehensive analysis in conjunction with clinical and imaging characteristics [10–12].

## 3. Statistical Analysis

We used SPSS version 25 (IBM SPSS) to analyze the data statistically. Categorical variables and continuous variables were summarized as frequencies or percentages and are expressed as means ± standard deviations (SDs). Categorical variables were compared with Pearson's *χ*^2^ test. A *p* value < 0.05 was considered significant [13].

## 4. Results

### 4.1. Clinical Characteristics of Participants

The data for 77 patients with long COVID and 52 diagnosed with both long COVID and *M. pneumoniae* infection were analyzed. All patients suffered mild acute COVID. The total number of long COVID patients included in the study was 129, 52 (40.31%) of whom were men and 77 (59.69%) were women. The mean age of the patients was 42.89 ± 15.89 years (ranging 16–88 years). The two groups did not differ significantly in terms of age (*p* > 0.05) but differed significantly in the distribution of sex (*p* < 0.05). Female long-COVID patients were more frequently infected with *M*. *pneumonia* than male patients (odds ratio (OR) = 2.28). Long COVID patients coinfected with *M. pneumoniae* were more likely to have symptoms such as cough, sputum production, palpitation, chest tightness, shortness of breath, or chest pain than were those not infected with *M. pneumonia*, but these differences were not statistically significant (*p* > 0.05). Thirty-six (27.90%) patients enrolled in the study also suffered chronic conditions, such as asthma, hypertension, diabetes mellitus, anemia, and atrial fibrillation. However, there was no meaningful difference in comorbidities between the two groups (Table 1).

### 4.2. Comparisons of Laboratory Results of Long COVID Patients With or Without *M. Pneumonia* Infection

A comparison of the laboratory test results of the long COVID patients and those coinfected with *M. pneumoniae* indicated that the white blood cell (WBC), red blood cell (RBC), hemoglobin, creatine kinase, and C-reactive protein levels did not differ significantly between the two groups. The patients with only long COVID had almost normal laboratory parameters. However, increases in aspartate transaminase (AST), and alanine transaminase (ALT) were observed more frequently in long COVID patients without *M. pneumoniae* infection (Table 2).

### 4.3. Chest CT Findings

**Figure 1:**
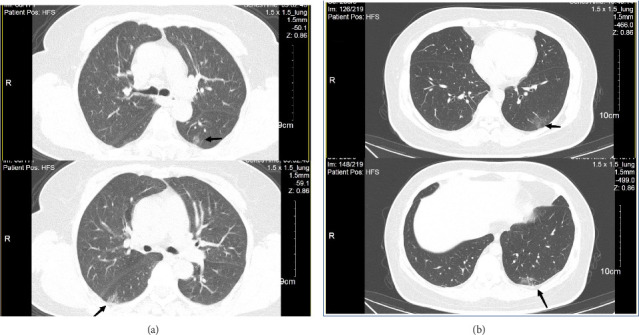
(a) The CT examination of long COVID patients with *M*. *pneumoniae* infection demonstrated a GGO in the dorsal outer zone of the bilateral lung (arrowhead); (b) the CT examination of long COVID patients with *M. pneumoniae* infection showed a GGO in the dorsal outer zone of the left lower lobe (arrowhead).

Fourteen patients were not examined with CT. Fifty-five patients in the long COVID group (55/70, 78.57%) had positive CT findings, whereas 60% (27/45) of long COVID patients coinfected with *M. pneumoniae* had positive imaging findings. These findings did differ significantly (*p*=0.032). Abnormal imaging was more commonly seen in the of long COVID patients than in the long COVID patients coinfected with *M. pneumoniae* (OR = 2.44). Ground-glass opacity (GGO) distributed in the dorsal outer zone was the predominant abnormality detected in these patients (Figure 1) [14].

## 5. Discussion

Social distance keeping and mandatory mask-wearing have led to a significant decrease in the incidence of various respiratory infectious diseases, including *M. pneumoniae* and influenza. Because of the lifting of COVID-19 restrictions, China's epidemiological data on *M. pneumoniae* are increasing. Coinfections with coronaviruses and other respiratory pathogens have frequently been observed since the lifting of COVID-19 restrictions. Several studies of these coinfections have been reported. Lansbury et al. [15] detected a bacterial coinfection in 7% of hospitalized COVID-19 patients, and *M. pneumonia* was the most common bacterium involved. Tang et al. [16] reported that coinfection correlated with elevated procalcitonin (PCT) levels in patients with COVID-19 pneumonia, which was successfully treated with anti-inflammatory therapies. However, there have been few comprehensive studies of long COVID patients with coinfections.

### 5.1. Risk Factors of Long COVID With *M. pneumoniae* Coinfection

In this study, a high proportion (42.3%) of long COVID patients was coinfected with *M. pneumonia*, and coinfection was detected significantly more often in women than in men. Several papers have suggested that underlying immune-system disorders cause the persistent symptoms of long COVID. High levels of autoantibodies have been found in some patients with COVID-19 more generally. Several studies have indicated that low or no production of SARS-CoV-2 antibodies and other insufficient immune responses during the acute phase of COVID-19 can predict the development of long COVID [17, 18].The innate and adaptive immune responses could be the essential factors making females more susceptible to long COVID [19].Women being more likely to have lower levels of antibodies and being less likely to seroconvert overall [20].

The primary clinical symptoms of long COVID are fatigue, chest pain, dyspnea, palpitations, arthralgia, muscle pain, cognitive impairment, and anxiety. In recent studies, fatigue was the most widespread symptom in long COVID patients. And cognition-related symptoms and cardiovascular complications were also reported in the latest international survey [3]. Because our data were all collected from respiratory clinic patients, respiratory dysfunction was recorded most frequently. Cough was the main symptom for which long COVID patients visited the clinic, followed by expectoration, shortness of breath, and chest tightness. These respiratory symptoms may be related to virus-induced changes in lung volume and airway clearance. Patients with *M. pneumoniae* also reported nonspecific respiratory symptoms, including general malaise and dry cough. The presence of *Mycoplasma* coinfection could be easily overlooked due to the similar presentations [21].

Koc et al. [9] reported that bronchial asthma, type 2 diabetes, and psychological disorders increase the risk of long COVID. Patients with hypertension, diabetes, and anxiety were also included in the present study. The risk of long COVID coinfected with *M. pneumoniae* was not increased by comorbidities.

### 5.2. Clinical Indicators in Long COVID Patients Coinfected With *M. pneumoniae*

Yong et al. [22] reported that patients with long COVID had higher levels of C-reactive protein, leukocytes, lymphocytes, and lactate dehydrogenase than those without long COVID. However, we detected the expected levels of leukocytes, erythrocytes, platelets, hemoglobin, C-reactive protein, lactate dehydrogenase, creatine kinase, and CK-MB in both long COVID patients with and without *M. pneumoniae* coinfection. The significant deviations from normal observed in inflammatory and vascular biomarker levels may be attributable to the severity of the COVID infection. The patients enrolled in our study were all mildly ill. A low viral load is likely to trigger a lower grade inflammatory response in the acute phase of viral infection, resulting in less damage to the host during long COVID.

### 5.3. Imaging Findings of Long COVID Patients Coinfected With *M. pneumoniae*

CT is the most widely used imaging technology for respiratory diseases [23, 24]. The chest CT manifestations in adults with COVID-19 or *M. pneumoniae* are the interstitial changes [25]. GGO, consolidation, linear opacity, vacuolar sign, and a crazy-paving pattern are common pulmonary lesions in COVID-19 patients. In contrast, the lesions in *M. pneumonia*–infected patients are predominantly distributed along the bronchi, and the bronchial wall is thickened, accompanied by tree buds/fog signs [26]. Our study showed that in patients with long COVID with or without *M. pneumoniae* infection, the major imaging change was GGO, distributed in the dorsal outer zone. *M. pneumoniae* infection is not considered to pose a risk of lung lesions in patients with long COVID. However, Bazdar et al. [27] reported 29 different imaging findings in long COVID patients, classified as interstitial (fibrotic), pleural, airway, and other parenchymal abnormalities. While CT imaging can provide valuable insights into the physiological effects of long COVID, the condition's presentation can be highly variable among patients. Imaging findings should be interpreted with clinical symptoms and other diagnostic information. To properly evaluate long COVID, more research is required into the lung imaging findings of these patients [28].

## 6. Conclusions

In this study, we detected no association between *M. pneumoniae* coinfection and biochemical abnormalities in patients with long COVID. Female long COVID patients and those with normal radiography were significantly more likely to present with *M. pneumoniae* coinfection. No correlation was detected between *M. pneumoniae* coinfection in long COVID patients and their pre-existing conditions, age, or symptoms. Various guidelines focus on the treatment and management of COVID-19, but there is little information on long COVID in these guidelines [29]. Based on the results of our study, the clinical symptoms of the syndrome were the primary health problem, and additional antibiotics for *M. pneumoniae* coinfection may be not necessary. Improving the clinical characterization of long COVID may be critical to the provision of adequate treatment options.

## Figures and Tables

**Table 1:** The difference in clinical characteristics between long COVID patients and MP coinfected patients.

	Total	Long COVID	Long COVID coinfected MP	*p* value
Patients (*n*)	129	77	52	
Sex				0.029
Male	52	37	15	
Female	77	40	37	
Age (years)				0.056
≤ 20	2	1	1	
21–40	67	40	27	
41–60	37	17	20	
> 61	23	19	4	
Symptoms				
Cough	120	74	46	0.095
Expectoration	80	50	30	0.406
Palpitation	10	6	4	0.983
Chest pain	7	3	4	0.351
Chest tightness	35	23	12	0.395
Shortness of breath	27	15	12	0.622
Chronic disease	36	23	13	0.205
CT scan result				0.032
Ground-glass opacity (GGO)	82	55	27	
No obvious abnormalities	33	15	18	

**Table 2:** The difference in laboratory tests between long COVID patients and MP coinfected patients.

	Total	Long COVID	Long COVID coinfected MP
WBC (10^9^/L)	6.81 ± 1.84	6.92 ± 1.92	6.63 ± 1.71
RBC (10^12^/L)	4.66 ± 0.50	4.73 ± 0.50	4.56 ± 0.48
PLT (10^9^/L)	246 ± 57	242 ± 51	251 ± 64
Hb (g/L)	135 ± 17	139 ± 16	134 ± 16
CRP > 10 mg/L	2.77 ± 2.86	2.78 ± 2.50	2.76 ± 3.54
LDH (U/L)	195 ± 41	198 ± 45	191 ± 31
CK (U/L)	86 ± 38	92 ± 41	75 ± 29
CK-MB (ng/mL)	2.05 ± 0.83	2.06 ± 0.69	2.03 ± 1.07
ALT (U/L)	29 ± 21	32 ± 24	22 ± 14
AST (U/L)	26 ± 11	29 ± 13	21 ± 3

## Data Availability

Data are available on request.
